# HYFIS vs FMR, LWR and Least squares regression methods in estimating uniaxial compressive strength of evaporitic rocks

**DOI:** 10.1038/s41598-023-41349-1

**Published:** 2023-08-29

**Authors:** Mohamed Yusuf Hassan, Hasan Arman

**Affiliations:** 1 Department of Statistics, College of Business, United Arab Emirates University, P.O. Box: 15551, Al Ain, United Arab Emirates; 2 Department of Geosciences, College of Science, United Arab Emirates University, P.O. Box: 15551, Al Ain, United Arab Emirates

**Keywords:** Civil engineering, Environmental sciences

## Abstract

The uniaxial compressive strength (UCS) of the rock is one of the most important design parameters in various engineering applications. Therefore, the UCS requires to be either preciously measured through extensive field and laboratory studies or could be estimated by employing machine learning techniques and several other measured physical and mechanical explanatory rock parameters. This study is proposed to estimate the UCS of the evaporitic rocks by using a simple, measured point load index (PLI) and Schmidt Hammer (SHV_RB_) test rock blocks of evaporitic rocks. Finite mixture regression model (FMR), hybrid fuzzy inference systems model (HYFIS), multiple regression model (MLR), and locally weighted regression (LWR) are employed to predict the UCS. Different algorithms are implemented, including expectation–maximization (EM) algorithm, Mamdani fuzzy rule structures, Gradient descent-based learning algorithm with multilayer perceptron (MLP), and the least squares. Coefficient of Determination (R^2^), Root Mean Square Error (RMSE), Mean Absolute Error (MAE) and A20-index accuracy measures are used to compare the performances of the competing models. Based on all the above measures, LWR outperformed with the other models whereas the HYFIS model has a slight advantage over the other two models.

## Introduction

The evaporitic rocks are highly sensitive either on the large (km) or small (m) scales to anthropogenic distortions such as groundwater level changes, intensive landscaping, watering and dewatering, infrastructure building, etc. due to different textures and structures. These changes could drastically affect the physical and the mechanical characteristics of evaporitic rocks. Therefore, precise measurement and highly accurate prediction of the engineering parameters of the evaporitic rocks is critically important in several engineering disciplines, including geotechnical, mining and geological^1^.

Correct measurements of those parameters need to carry out intensive and careful works, and to perform various physical and mechanical tests by following internationally recognized standard testing procedures either in the field or in the laboratory^2–4^. Obtaining such an accurate engineering parameter is highly expensive, time-consuming, and requires patience and vigilant works. However, those parameters could be estimated by using different machine learning techniques, machine learning algorithms have gained more attention during last two decades in the field of data science. These techniques are used to estimate the parameters with the help of some other measured physical and mechanical parameters of the rocks that can be easily obtained.

The UCS, the point load index (PLI) and the Schmidt Hammer (SHV) are well-known standardized tests by the American Society for Testing and Materials^3–5^. and the International Society for Rock Mechanics^2^. They have been broadly recognized and used in measuring the strength of rock materials. However, the PLI and SHV which are faster, more economical and simpler tests compared to the UCS test in sample collection and in the testing procedures are commonly used. Furthermore, rock samples for the UCS test need careful preparation which is very expensive and requires tedious works to fit the standards of core sample specifications to attain valid test results, particularly, for weak rocks like evaporites.

Thus, the UCS could be estimated with simple statistical approaches using some mechanical and physical properties of rocks. In the literature. the PLI and SHV or both have been often used to estimate the UCS of various rocks^6–21^.

During the last two decades, a number of researchers have been proposing different estimating machine learning techniques to predict the UCS of different rock types. Alvarez Grima and Babuska^22^ used a fuzzy model, Takagi–Sugeno (TS), to estimate the UCS from various rock types of 226 rock samples. They compared their model with a multiple linear regression model and reported that the TS fuzzy model performed better than that of the multiple linear regression model. Gokceoglu et al.^23^ initiated some predictive models to estimate the UCS of some clay-bearing rocks using the slake durability index (SDI) and clay amount. They stated that the fuzzy interface model was slightly better than that of the regression models due to the flexibility of the fuzzy model. Yilmaz and Yuksek^24^ predicted the UCS and the modulus of elasticity (E) of gypsum using multiple linear regression, artificial neural networks (ANNs) and adaptive neuro-fuzzy inference system (ANFIS) models. They discovered that the ANFIS model provided higher accuracy prediction to estimate the UCS and the E. So, they suggested that the employed models may be used with a tolerable accuracy especially at the preliminary designing stage. Amin et al.^25^ employed a genetic algorithm (GA) as a heuristic search method to select the best transformation of the independent variables in the regression models to estimate the UCS and the E. Their results revealed that the GA models were more accurate than the multiple linear regression (MLR) and had better fit in terms of the formulation simplicity and the acceptable accuracy. Yesiloglu et al.^26^ predicted the UCS of granitic rocks from their mineral contents using the ANFIS, and their predictions were validated by nonlinear multiple regression model. The obtained results from their study indicated that both models were acceptable, but the ANFIS performed better than the multiple regression model in predicting granitic rock’s UCS. Majdi and Rezaei^27^ attempted to predict the UCS of various rocks implementing ANN and multivariable regression analysis (MVRA). They concluded that the ANN model was better than the MVRA. In addition, the SHV and rock density were the most effective parameters in predicting the UCS in based on the sensitivity analysis. Ceryan et al.^28^ tried to estimate the UCS of carbonate rocks using the Levenberg–Marquardt algorithm based on ANN model (LM-ANN) and compared with the MLR. Their results showed that the LM-ANN model was more accurate than the MLR in predicting the UCS. Beiki et al.^29^ evaluated the applicability of the genetic programing (GP) in prediction of the UCS and E of carbonate rocks. They found that the GP models were acceptable for the prediction of the UCS and the E of the carbonate rock than that of the regression models especially when multiple error criteria is used. Torabi-Kaveh et al.^30^ proposed MLR, multiple nonlinear regression (MNL) and ANNs to predict the UCS and the E using physical properties of limestones. Their study indicated that ANN models were better than that of the other models in estimating the UCS and the E of limestones. Mohamad et al.^31^ investigated the possibility of a hybrid particle swarm optimization (PSO)-based ANN model application for the prediction of soft rocks (mostly shale) of the UCS. Their results revealed that the investigated model fitted well and provided high performance indices for the prediction of the UCS. Armaghani et al.^32^ formulated how the UCS and the E of granite could be predicted using ANFIS. They concluded that the predictive ANFIS model outperformed than those of the MRA and ANN models. Armaghani et al.^33^ also tried to predict the UCS of sandstone using different modelling techniques such as simple linear regression, MLR, MNR, ANN and ICA-ANN. They found that the ICA-ANN model was the best model compared the other models, and they suggested that the ICA-ANN model must be used for the prediction of the UCS from similar rock type with caution. Ferentinou and Fakir^34^ developed a back propagation ANN model to estimate the UCS of some sedimentary and igneous rocks. They concluded that the developed approach was effective in estimating the UCS. Fattahi^35^ demonstrated the use of various modelling techniques of support vector regression (SVR) optimized by artificial bee colony algorithm (ABC) and ANFIS-subtractive clustering method (SCM) (ANFIS-SCM) for predicting the UCS of rocks from the SHV values. His study showed that the ANFIS-SCM model was the best model to predict the UCS of rocks from the SHV values with high precision. Heidari et al.^36^ proposed simple linear regression model, MLR and the Sugeno-type fuzzy algorithm for the prediction of the UCS of some sedimentary rocks. Their study revealed that both MLR and fuzzy inference systems were better than that of the simple regression model to predict the UCS. However, they have mentioned that the fuzzy inference systems were much better that of the other models. Wang et al.^37^ applied a random forest (RF) predictive model for estimating the UCS of rocks by utilizing data collected from previous research and using simple index tests. Laboratory tests were performed to check the validity of the predictive model results, and they have suggested that the random forest (RF) predictive model could be used to predict the UCS of rocks from the measured values of the rock mechanics and engineering geology. Rezai and Asadizadeh^38^ focused applying on a new hybrid intelligent model, including ANFIS, GA and PSO for the prediction of the UCS of the weak to the very strong rock types. Their study showed that the ANFIS-GA provided relatively better accuracy than that of the ANFIS-PSO, but both models were better than that of the MLR. Nasiri et al.^39^ presented the Shapley Additive Explanations (SHAP) which is one of the most recent explainable artificial intelligent (XAI) models for the prediction of the USC and the E of the travertine. Their results revealed that the accuracy of the SHAP-XGBoost model was higher than that of the other competitive models, including RF and SVR. Therefore, XAI could be used to analyze complicated problems in rock mechanics.

In earlier studies, either empirical or predictive models like least squares regression techniques, adaptive neuro-fuzzy inference system, artificial neuron networks, genetic algorithm, imperialist competitive algorithms and others with various measured mechanical and physical parameters of the rocks has been used to predict UCS for different rock types. However, we are not aware of any study in the literature that has compared the performance of finite mixture regression (FMR), hybrid fuzzy inference systems (HYFIS), locally weighted regression LWR and multiple regression (MLR) methods for the prediction of the UCS values based on the available literature. Thus, this study aims to develop the best predictive models from the above-mentioned methods to estimate the UCS of the evaporitic rocks from the simple measured parameters of the PLI and SHV_RB_. Such an approach, especially during a preliminary design stage of any engineering structures, could be faster and economical if different laboratory test results indicate variations. On the other hand, although machine-learning techniques are powerful in dealing with non-linear systems, but they need large enough data set that can represent the system to be investigated.

## Sampling site and experimental framework

**Figure 1:**
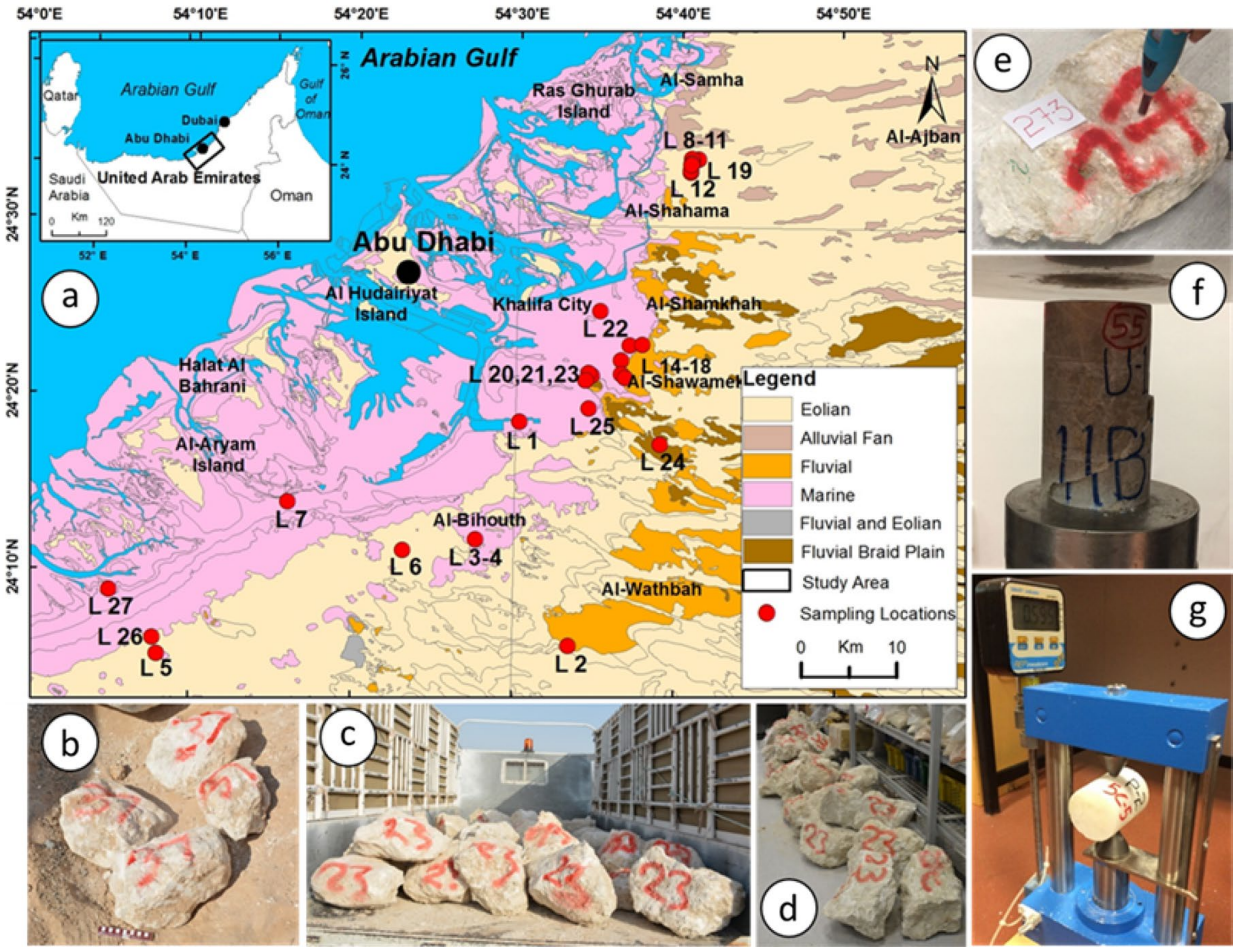
(**a**) Geological map of the Abu Dhabi and sampling locations (generated with ArcGIS 10.8^40^), (**b**) rock block samples, (**c**) transportation of rock block samples, (**d**) storage of rock block samples in the laboratory, (**e**) Schmidt hammer test on rock block samples (SHV_RB_), (**f**) uniaxial compressive (UCS) test, (**g**) point load index (PLI) test.

Representative evaporitic rock blocks, 152, were collected from 27 locations from Abu Dhabi city and its surrounding areas (Fig. 1a,b). Evaporitic rock blocks were carefully inspected and those without visible defects such as cracks, fractures, alteration zones were transported to a laboratory and stored under the laboratory condition (Fig. 1c,d). Before coring, 139 sets of Schmidt hammer tests were conducted on both side of evaporitic rock blocks (SHV_RB_) by following the suggested ASTM standards (Fig. 1e). The UCS and PLI test samples were cored from 108 and 138 suitable evaporitic rock blocks by following the suggested ASTM and ISRM standards. 257 and 327 NX size core samples for the UCS and the PLI tests were prepared and the UCS and the PLI tests were conducted on intact rock core samples according to suggested tests standards (Fig. 1f,g). If the performed tests for the UCS and the PLI did not fulfill the required specifications of the suggested standards due to either core sample features or rock failing unexpectedly along the existing invisible weakness plane, those test results were excluded in the analyses.

## Methodology

**Figure 2:**
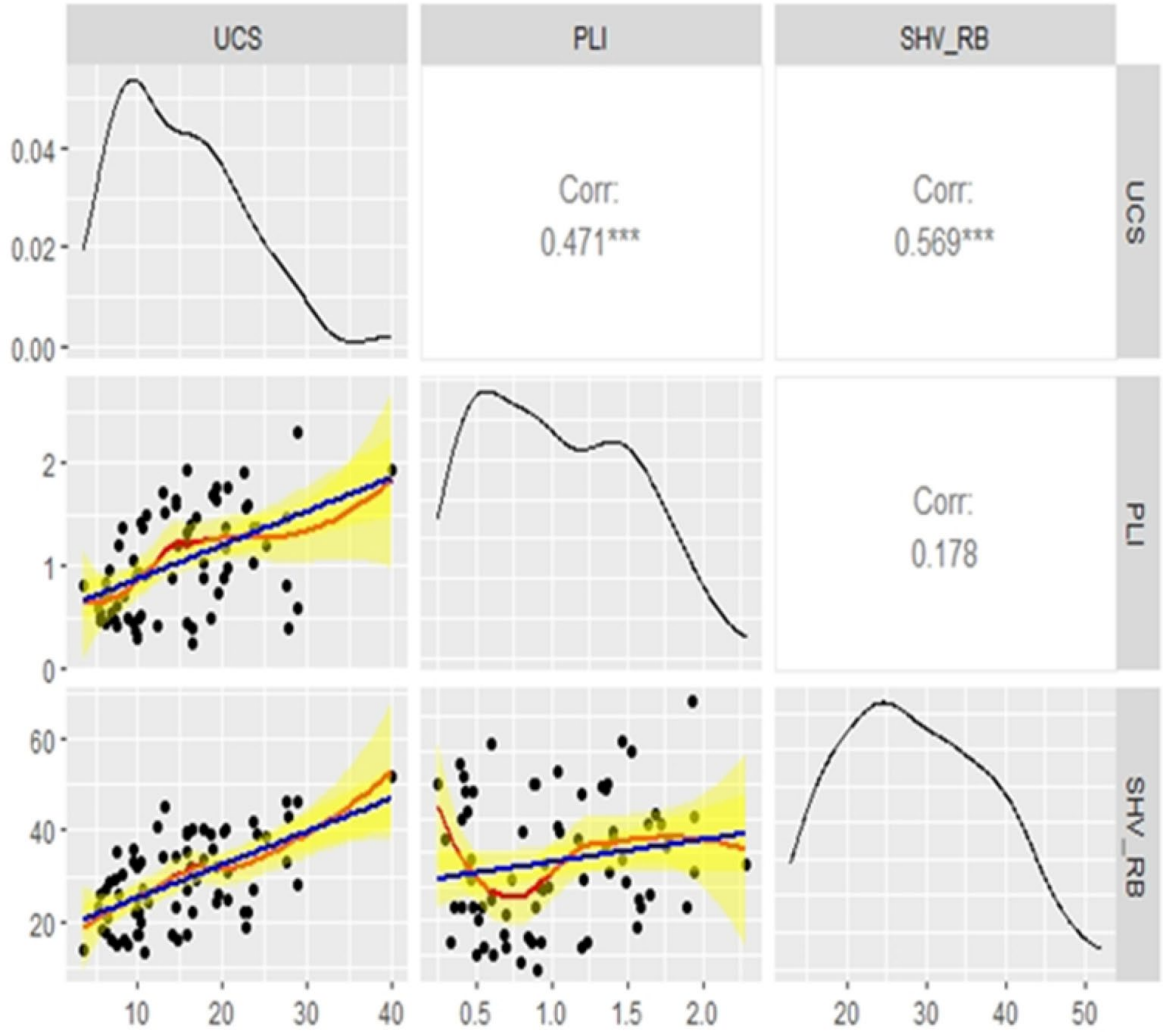
Scatterplots and probability density plots.

**Figure 3:**
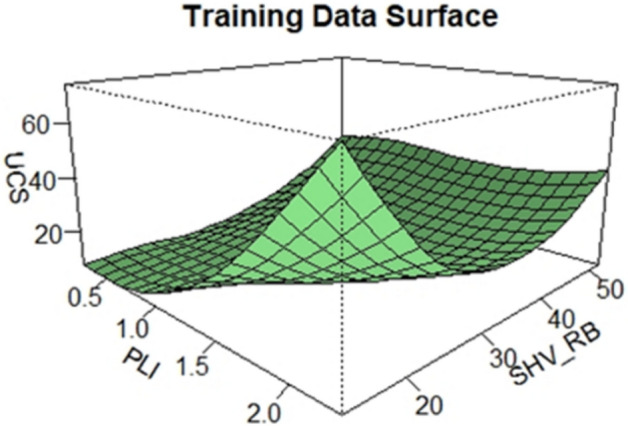
Surface plot of the data.

After the samples were collected from the rocks and tested, best predictors of the UCS were searched from a pool of physical and mechanical variables by using their correlations with the UCS. Based on this search, PLI and SHV_RB_ are chosen as the best predictors of the UCS, and then a qualitative and quantitative assessments of the three variables, UCS, PLI and SHV_RB_, were conducted. Visualization tools like histograms, density plots, Q-Q plots and surface plots are displayed to investigate the relationships of the variables. These tools are some of the best empirical methods and visually appealing approaches to explore the shape and the underline distribution of a given data^41^. As can be seen in Figs. 2 and 3, both the density and the surface plots show that the relationship between the UCS and PLI is clearly non-linear, but UCS and SHV_RB_ are linearly related. Besides that, the density plots of the UCS and PLI show bimodality whereas that of the SHV_RB_ seems unimodal. Since the departure from unimodality has many implications in data analysis, one way to investigate the shape of an underline distribution other than the above tools is to conduct former goodness of fit tests. Excess Mass Test, introduced by Müller and Sawitzki^42^ is used to test the unimodality of the UCS distribution, this test is one of the well-known goodness of fit tests that can be used to test multimodality. A test statistic of 0.088201 with a p-value of 0.134 is obtained which shows that the distribution does not deviate from unimodality, and the hypothesis of bimodality is not supported at the 5 percent significance level.

Descriptive statistics of the data, including 95% confidence intervals for the means, is summarized in Table 1.

**Table 1:** Descriptive statistics of the data.

Variable	Number	Mean	Standard deviation	Median	95% CI for μ
UCS	73	14.87	7.31	14.46	(13.17, 16.57)
PLI	73	1.04	0.51	0.94	(0.92, 1.15)
SHV_RB_	73	28.72	9.29	27.00	(26.55, 30.89)

## Model development

The aim of this study is to compare the quality of prediction for four statistical and machine learning techniques in estimating and capturing the nature of UCS by using the variations of the PLI and SHV_RB_. The competing models are FMR, HYFIS, MLR, and LWR. Those models are well-known for their high accuracy in the modeling and the prediction of quantitative datasets.

### Finite mixture regression model (FMR)

The FMR is used to model heterogeneous data that have multiple modes. This type of data generally contains several sub-populations that depend on some covariates that need to consider separately before their outcomes are merged to find unique prediction results for the general population. This technique is first introduced by Quandt and Ramsey^43^, and it can handle missing data and capture the structure of the process being modeled. These models provide a new set of models for modeling heterogenous data as well as homogenous data. De Veaux^44^ established an EM approach to fit those regression models. The EM algorithm has some limitations, including reproducibility. Nevertheless, FMR models provide high accuracy predictions and a new set of models for modeling both heterogenous and homogenous datasets if it is handled with care and there is a solid knowledge of the domain^45,46^.

Let **Y** be a vector of a dependent variable that is linearly related to k vectors of explanatory variables **X**_**1**_**, X**_**2**_**, …, X**_**k**_ through vectors of parameters **β**_**1**_**, β**_**2**_**…, β**_**k**_. If **Y** contains several subpopulations and **X** = (**X**_**1**_**, X**_**2**_**, …, X**_**k**_ ) is the matrix of the explanatory variables. We say that **Y** is generated by the finite mixture regression **(FMR)** if it can be modelled as follows:

1 $$\phi \left(y|x;\varphi \right)=\sum_{k=1}^{K}{\alpha }_{k}{\phi }_{k}\left({y}_{i}|{x}_{i},{\varphi }_{k}\right)$$

where ${\phi }_{k}$ is a Gaussian density function of the ***k***^***th***^ component, ${y}_{i}$= ${x}_{i}^{T}{\beta }_{k}+{\epsilon }_{ik}$ for *i* = *1, 2,…,n, k* = *1,2,…, K*, and ${\beta }_{k}$ is a vector of regression parameters. ${\varphi }_{k}=({\beta }_{k}, {\sigma }_{k}^{2}$), $\varphi =$ ( ${\alpha }_{k}$ , ${\varphi }_{k}$), $\sum_{k=1}^{K}{\alpha }_{k}=1,$ ${\alpha }_{k}>0, k=1, 2, \dots , K.$

### Estimation of finite mixture regression by EM algorithm

The general EM algorithm^47^ is used to estimate the parameters of the mixture models. Let **Z = (Z**_**1**_**, …, Z**_**n**_**)** be unobservable random variables, where **Z**_**i**_** = (z**_**i1**_**, …, z**_**ik**_**)** is a ***p-***dimensional indicator vector and **z**_**ik**_ is unity if y_i_ comes from component ***k*** and zero otherwise. Now given all the data and assuming that Z’s and **X** are independent and the Z_i_ are independent of each other, the (conditional) log-likelihood function of the whole data can be written as follows:

2 $$L\left(\mathrm{\varphi }\right) = \sum_{\mathrm{i}=1}^{\mathrm{n}}\sum_{\mathrm{k}=1}^{\mathrm{K}}{\mathrm{z}}_{\mathrm{ik}}\mathrm{log}{\mathrm{\alpha }}_{\mathrm{k}}+\sum_{\mathrm{i}=1}^{\mathrm{n}}\sum_{\mathrm{k}=1}^{\mathrm{K}}{\mathrm{z}}_{\mathrm{ik}}\mathrm{log}{\upphi }_{\mathrm{k}}({\mathrm{y}}_{\mathrm{i}}|{\mathrm{x}}_{\mathrm{i}};{\varphi }_{k})$$

Once the log-likelihood function is obtained, the EM algorithm is implemented as follows:

**E Step:** Suppose that ***β = (β***_1_*,…, β*_*k*_*)*, ${{\varvec{\sigma}}}^{2}$**=** *(*${\sigma }_{1}^{2}$,…, ${\sigma }_{k}^{2}$*),* and ***α*** = *(α*_1_*,…,α*_*p*_*)* are known. Then the missing quantities **Z** are replaced by their conditional expectations, conditioned on the parameters and on the observed data ***(X, Y).*** The conditional expectation of the *k*^th^ component of **Z**_*i*_ is just the conditional probability that the observation y_i_ comes from the k^th^ component of the mixture conditioned on the parameters and the observed data. Let the conditional expectation of the *k*^th^ component of **Z**_*i*_ be ${\mathrm{E}}_{\mathrm{ik}}$. Then ${\mathrm{E}}_{\mathrm{ik}}$ = ${\alpha }_{k}{\phi }_{k}\left(y|x,{\varphi }_{k}\right)$/($\sum_{\mathrm{i}=1}^{\mathrm{K}}{\mathrm{\alpha }}_{\mathrm{i}}{\upphi }_{\mathrm{i}}(y|\mathrm{x};{\varphi }_{k})$ ).

**M step:** Suppose that the missing **Z**_***i***_’s are now known. The estimates of the parameters ***β,*** ${{\varvec{\sigma}}}^{2}$, and ***α*** can then be obtained by maximizing the log-likelihood function *L* in (2). The final estimates of the parameters are then obtained by iterating these two steps until convergence, for example, the estimates of the mixing proportions are computed as follows:

3 $${\mathrm{Z}}_{\mathrm{ik}}=\sum_{k=1}^{K}{\mathrm{E}}_{\mathrm{ik}}/(\sum_{\mathrm{i}=1}^{\mathrm{n}}\sum_{\mathrm{k}=1}^{\mathrm{K}}{\mathrm{E}}_{\mathrm{ik}})$$

### Hybrid neural-fuzzy inference system (HYFIS)

**Figure 4:**
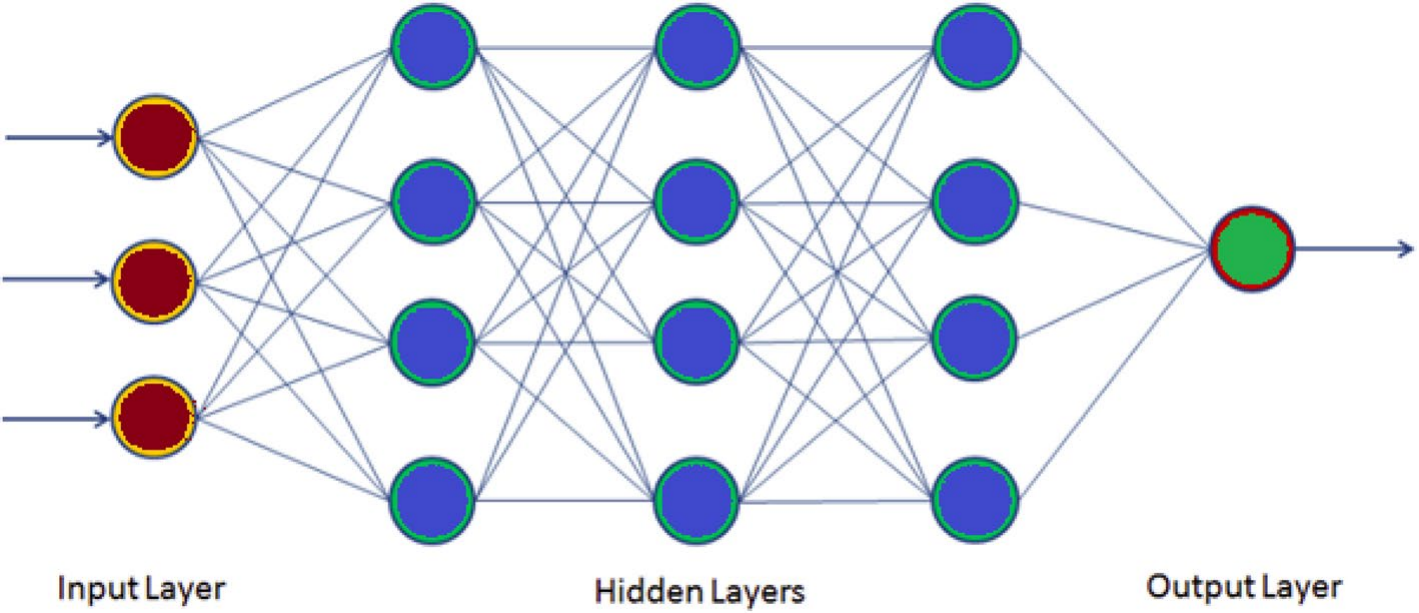
The structure of a HYFIS model with one input layer, three hidden layers and one output layer.

The HYFIS learning procedure was proposed by Kim and Kasabov^48^. It is one of the variant methods of fuzzy neural networks (FNN; Buckley and Hayashi^49^), this group is commonly known as the neuro-fuzzy systems, and they are widely used in machine learning. Those systems include some of the most popular hybrid machine learning techniques like adaptive neural networks fuzzy systems (ANFIS), FNN is a hybrid technique that combines artificial neural networks (ANN) with fuzzy rule-based systems (FRBSs), fuzzy rule-based systems are well-known techniques in soft computing. HYFIS is implemented by laid upon its ANN structure by FRBS rules, so the learning algorithm of the ANN adapts the FRBS parameters of Mamdani^50^ and Takagi and Sugeno Kang^51^. Several different schemes and architectures of this hybrid system have been proposed, such as fuzzy-logic-based neurons^52^, fuzzy neurons^53^, neural networks with fuzzy weights^49^, neuro-fuzzy adaptive models^54^. The HYFIS technique uses the Mamdani model as its rule structure, it has two phases for learning, the knowledge acquisition module and the structure and parameter learning. The knowledge acquisition module uses the techniques of^54^ whereas the learning of structure and parameters is a supervised learning method that use gradient descent-based learning algorithms with multilayer perceptron (MLP)^48^. The multilayer perceptron (MLP) is a connected class of feedforward artificial neural network (ANN) designed to approximate any continuous function; it can solve problems which are not linearly separable^55^, and it has three layers see Fig. 4—the input layer, output layer and hidden layer, which contains arbitrary number of hidden layers based on the given problem. The neurons of this algorithm use a nonlinear activation function like ReLU or the Sigmoid, this function generates a model that consists of a rule database and parameters of the membership functions. HYFIS uses the Gaussian function as a membership function, and it has two parameters which are optimized: its mean and variance. The predictions of the HYFIS can be performed by the standard Mamdani procedure.

### Locally weighted regression models (LWR)

The LWR is a technique of estimating a regression function **g(x)** through a multivariate smooth function. This procedure instead of estimating its parameters, it specifies computing of the fit at a target point **x**_**0**_ using only the regression of the nearby training observations. The method, which is a nonparametric, assumes that **g** is a parametric, smooth function. For every observation of the explanatory variables, a local neighborhood is chosen, then it fits the function to a vector of independent variables locally in way like the moving averages in time series. Let **y**_**i**_ for ***i = 1,…, n*** be ***n*** observations of the response variable, and let **x**_**i**_** = (x**_**i1**_**,…, x**_**ip**_**), i = 1,…, n**, be ***n*** observations with ***p*** predictors. Suppose that the data is generated by

4 $${{\varvec{y}}}_{{\varvec{i}}}=\mathbf{g}\left(\mathbf{x}\right)+{{\varvec{\varepsilon}}}_{{\varvec{i}}\boldsymbol{ }\boldsymbol{ }}\quad{\varvec{I}}\boldsymbol{ }=\boldsymbol{ }1\dots ,\boldsymbol{ }{\varvec{n}}$$

where $\mathbf{g}$ is a smooth function and **ε**_**i’s**_ are identically and independently normally distributed random variables with mean zero and variance ${{\varvec{\sigma}}}^{2}$**,** then ${{\varvec{y}}}_{{\varvec{i}}}$ is given by

5 $${{\varvec{y}}}_{{\varvec{i}}}={{\varvec{\beta}}}_{0}+{{\varvec{\beta}}}_{1}\left({{\varvec{x}}}_{{\varvec{i}}1}-{{\varvec{x}}}_{01}\right)+-\dots -{{\varvec{\beta}}}_{{\varvec{p}}}\left({{\varvec{x}}}_{{\varvec{i}}{\varvec{p}}}-{{\varvec{x}}}_{0{\varvec{p}}}\right)+{{\varvec{\varepsilon}}}_{{\varvec{i}}}$$

For computational and theoretical purposes, a weight function is defined so that only values within a smoothing window or a neighborhood of each value is included in that regression. A common choice of the weighting function is the following tri-cubic weight function.

6 $$T\left(u;t\right)=\left\{\begin{array}{c}(1-(\frac{u}{t}{)}^{3}{)}^{3},\, for \,0 \le u<t \\ 0, for\, u\,\ge t\end{array}\right.$$

Let ${\Delta }_{i}\left(x\right)=|x- {x}_{i}|$ be the values of these distances ordered from smallest to largest, and let *h* be the width of the window or the span. The weight function is defined as follows:

7 $${w}_{i}\left(x\right)=T\left({\Delta }_{i}\left(x\right);h\right)$$

Once the weights are carefully chosen, the LOESS method, which is based on the least square technique, is implemented by minimizing the following quadratic function.

8 $$\sum_{k=1}^{n}{w}_{k}({x}_{i})({y}_{k}-{\beta }_{0}-{\beta }_{1}{x}_{k}-\dots -{\beta }_{p}{x}_{p}{)}^{2}$$

Then the following estimate of $\mathbf{g}(\mathbf{x})$ is obtained.

9 $$\widehat{{\varvec{g}}}\left(x\right)=\sum_{i=1}^{n}{l}_{i}({\varvec{x}}){y}_{i}$$

Clearly, the loess estimate, $\widehat{{\varvec{g}}}\left({\varvec{x}}\right)$, is a linear combination of the ${{\varvec{y}}}_{{\varvec{i}}}$**,** where the ${{\varvec{l}}}_{{\varvec{i}}}$ depend on ${{\varvec{x}}}_{{\varvec{k}}}$ for **k = 1, …, n**, and W.

## Results and discussion

In this study, four machine techniques, including FMR, HYFIS, MLR and LWR, are used for the modeling and the prediction of the UCS by using 73 rock block samples collected from Abu Dhabi area, United Arab Emirates. Firstly, after the data are collected, a variable screening work based on the correlations between the UCS and each of the explanatory variables is performed to identify the best predictors of the UCS. Those relationships have shown that PLI and SHV_RB_ have the highest correlations with the UCS, and they are chosen to predict the UCS values. Secondly, the data were randomly split into training and test sets with a 70:30 ratio (70% training and 30% testing^56^), and thirdly, the two independent variables of the training data were standardized into z-scores. The standardization method is widely used to improve the convergence of machine-learning algorithms^57,58^. After data standardization, a ten-fold cross-validation (CV), which is a resampling method is used to validate the performance of a fitted model. When the models are trained, the performances of the four models from the test sample are compared using the results of the accuracy measures, coefficient of determination (R^2^), root mean square error (RMSE) and mean absolute Error (MAE) to determine the best model in predicting UCS.

### Estimation of Finite Mixture Regression by EM Algorithm

The best fitting FMR model for the prediction of the UCS is the second order model (K = 2) with a BIC and AIC values of 351.58 and 337.65 respectively.

10 $$\left(y|x;\varphi \right)=\sum_{k=1}^{2}{\alpha }_{k}{\phi }_{k}\left({y}_{i}|{x}_{i},{\varphi }_{k}\right)$$

**Figure 5:**
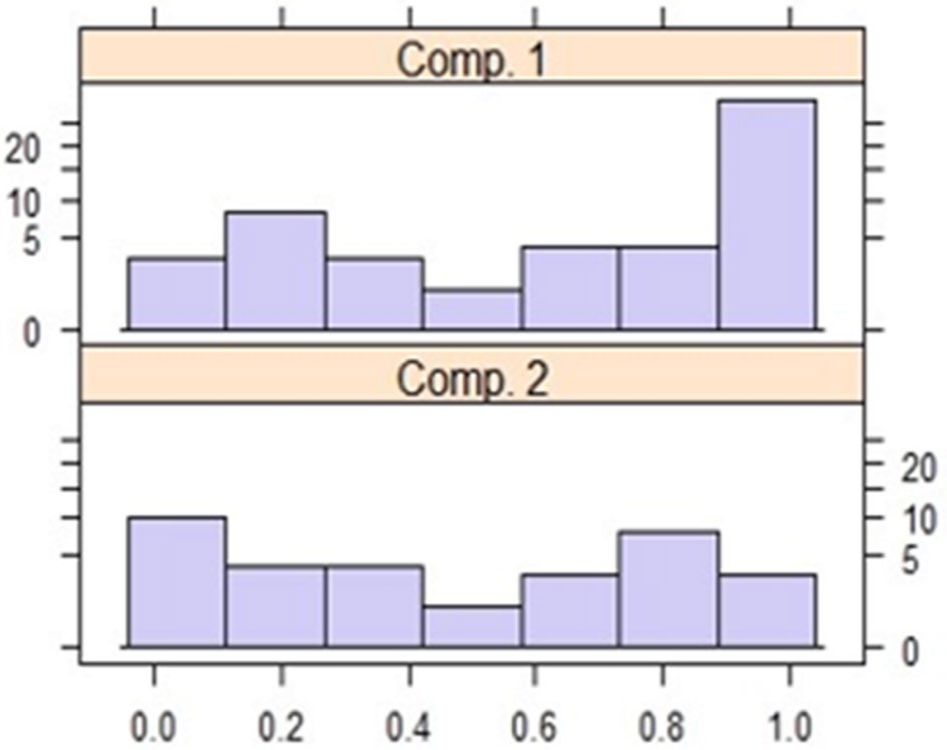
Rootogram of the posterior probabilities.

**Figure 6:**
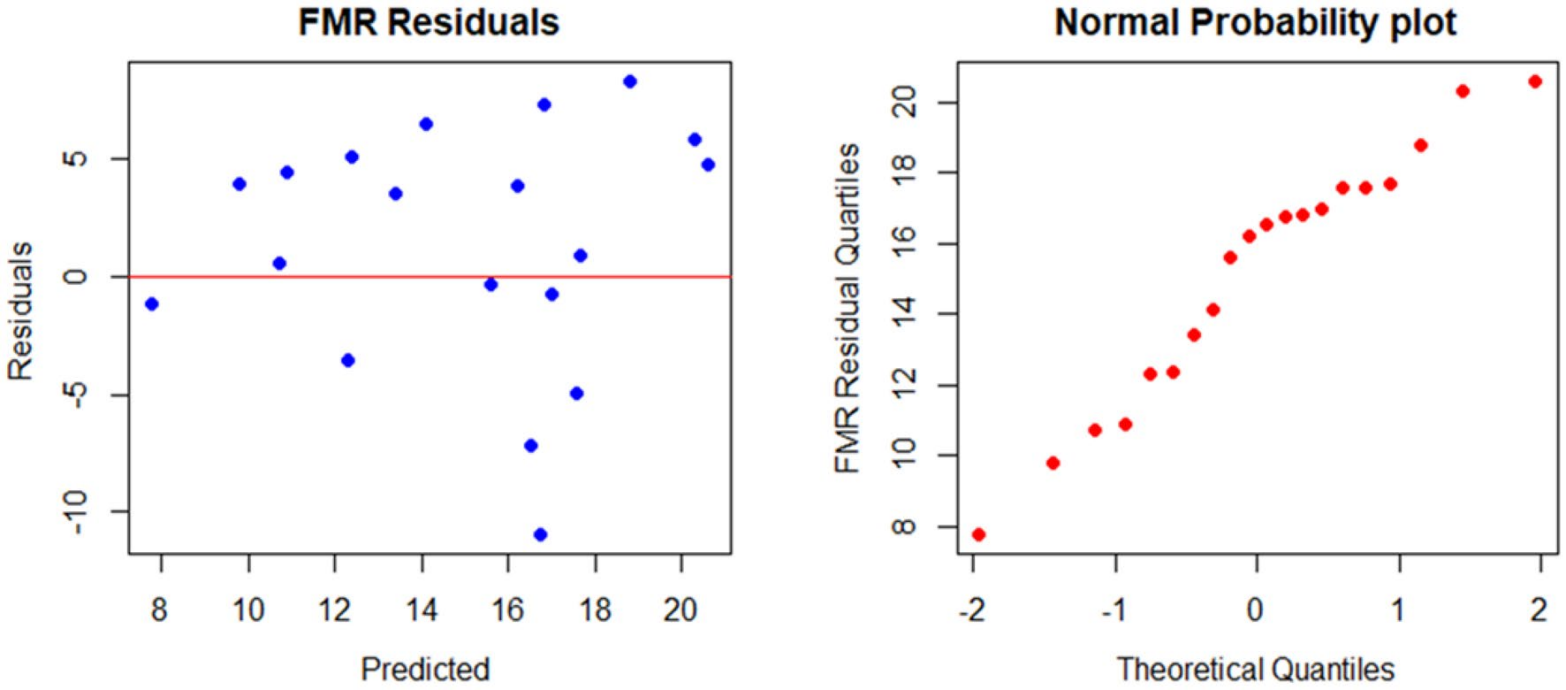
Residual plots of the FMR model.

The proportion estimates of the above model components are **α** = (α_1_, α_2_) = (0.737, 0.263) whereas the regression parameter estimates for the components are **φ**_**1**_ = (*β*_0,_ *β*_1_, *β*_2_) = (0, 4.67, 0.15) and **φ**_**2**_ = (*β*_0,_ *β*_1_, *β*_2_) = (0, 5.52, 0.410) respectively. The rootogram of the posterior probabilities for the fitted model is shown in Fig. 5. Since one of the EM algorithm limitations is reproducibility caused by initial values, the sensitivity of the algorithm to the initial values is examined by choosing several random starts and it converged to the same final estimates appearing in Table 2, which are all highly significant. Residual plots of this model are presented in Fig. 6, these plots do not show any deviations from normality.

**Table 2:** Finite mixture regression parameter estimates.

α = 0.737			
Component 1	Coefficients	Test statistic	P-value
PLI	5.52	3.74	P < 0.001
SHV_RB_	0.410	7.60	P < 0.001

1 − α = 0.263			
Component 2	Coefficients	Test statistic	P-value
PLI	4.67	2.58	P < 0.010
SHV_RB_	0.15	1.98	P < 0.047

### Hybrid neural-fuzzy inference system (HYFIS)

**Figure 7:**
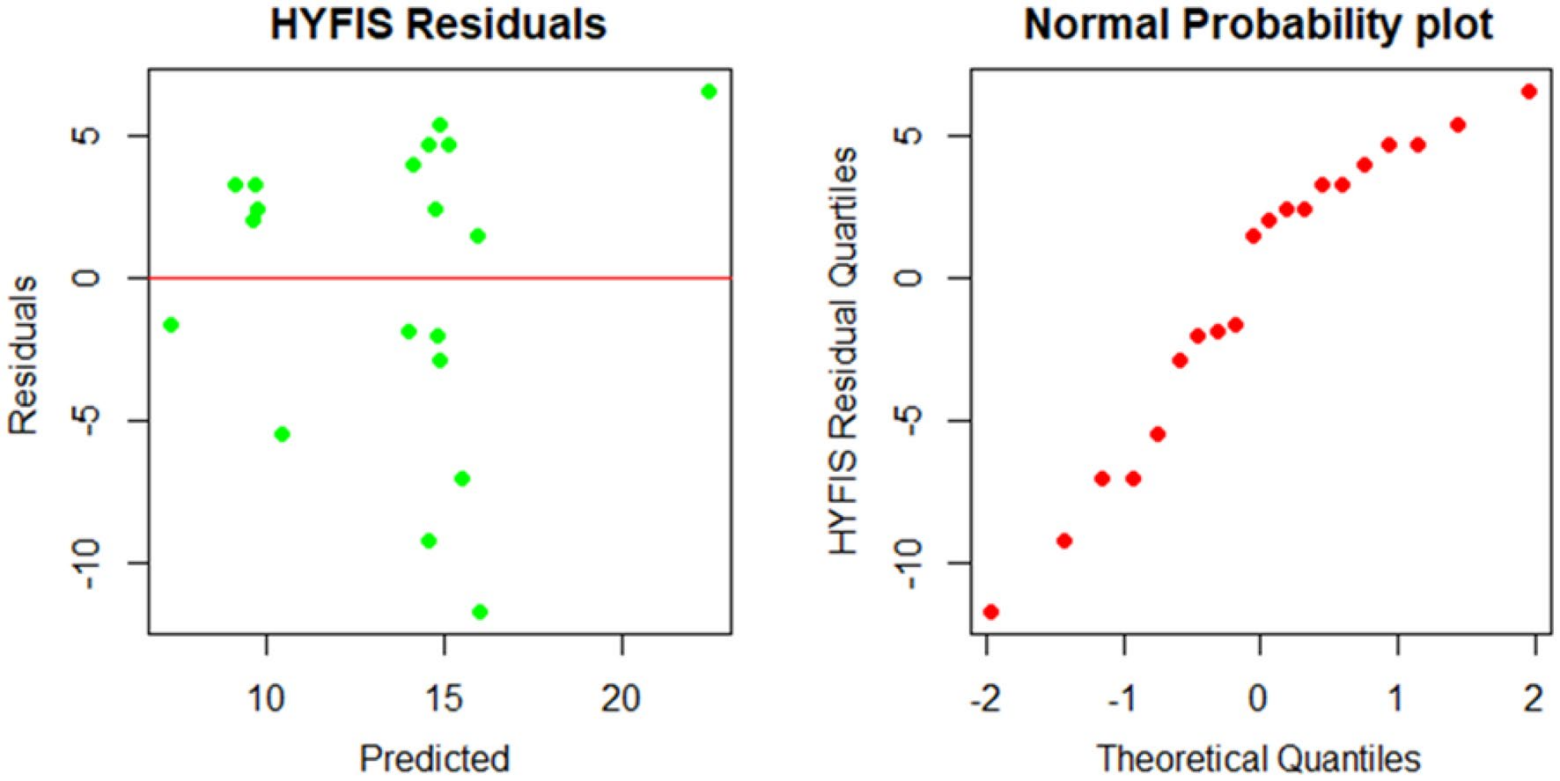
Residual plots of the HYFIS model. Color of dots.

The best HYFIS Mamdani fuzzy rule-based system (FRBS) model for the prediction of the UCS values is investigated using the two explanatory variables PLI and SHV_RB_. The best model chosen by the accuracy measures, the MAE, RMSE and the R^2^, is the model with gaussian membership functions, minimum t-norm, standard s-norm, modified COG defuzzification technique and Zadeh implication function. The training parameters of the best HYFIS model identified by the accuracy measures, RMSE and MAE, are three labels, 50 maximum iterations and a step size of 0.01. The residuals plots produced by the best fitted HYFIS model did not show any deviations from symmetry and heteroscedasticity pattern, see Fig. 7.

### Multiple linear regression model (MLR)

Regression variable selection procedures, including forward selection, backward elimination, and the best subsets are the most used methods to identify the best regression model.

**Figure 8:**
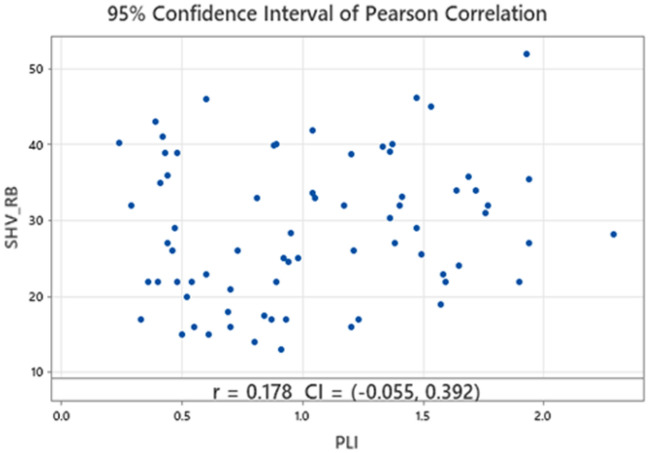
Correlation between PLI and SHV_RB_.

In this study, the best fitted regression model identified for the prediction of the UCS, using the training data, is the model with the two explanatory variables, PLI and SHV_RB_ with zero intercept. All the tests for the parameters were highly significant (see Table 3), and the variance inflation factor (VIF) of the model is very low (4.84) indicating that multicollinearity is not detected, VIF values more than 10 are considered to indicate serious multicollinearity. Besides that, the scatter plot of PLI vs SHV_RB_ on Fig. 8 shows a random pattern and a p-value of 0.178 for the Pearson correlation.

**Table 3:** MLR results.

Variable	Coefficient	T-statistic	P-value	VIF
PLI	4.53	1.390	P < 0.002	4.84
SHV_RB_	0.38	0.054	P < 0.001	4.84

**Figure 9:**
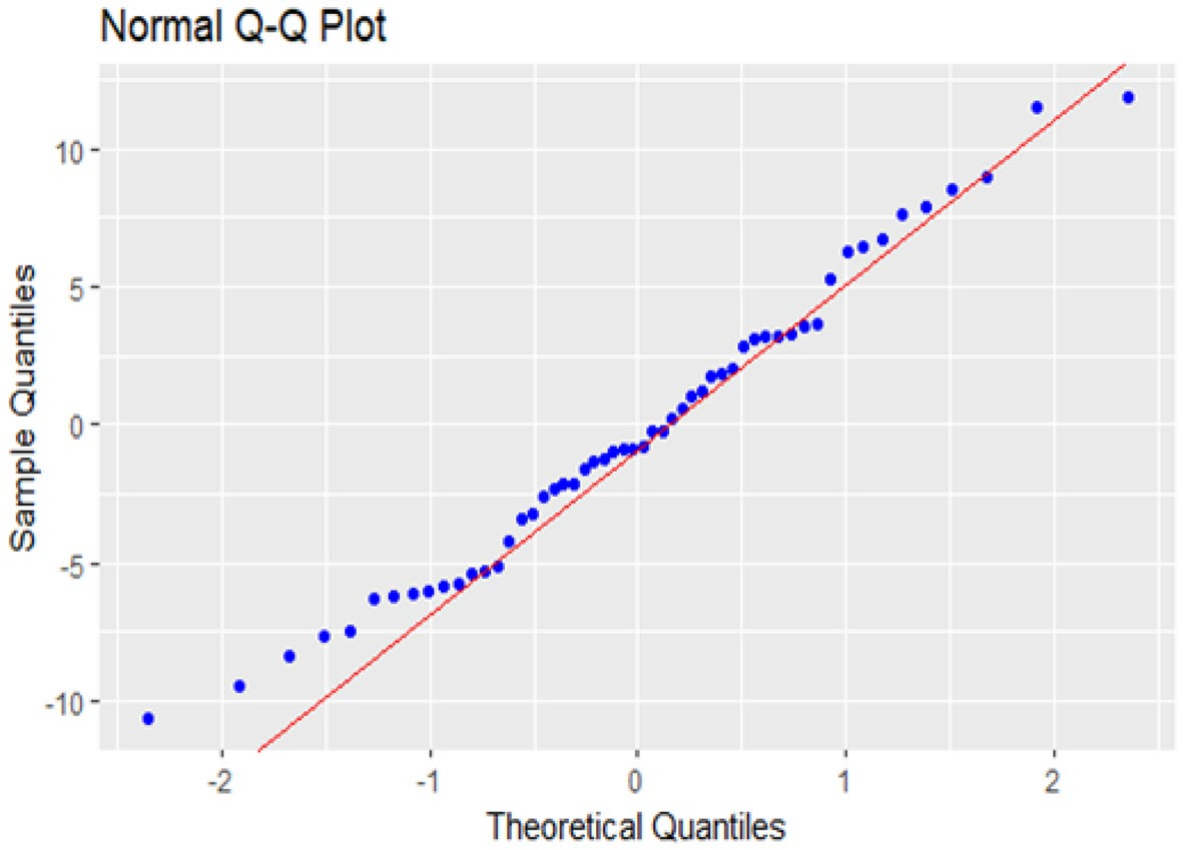
Normal probability quartiles.

**Figure 10:**
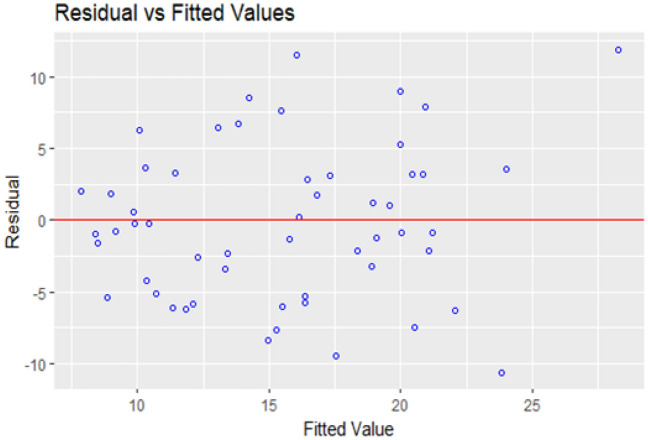
Fitted value vs Residuals.

**Figure 11:**
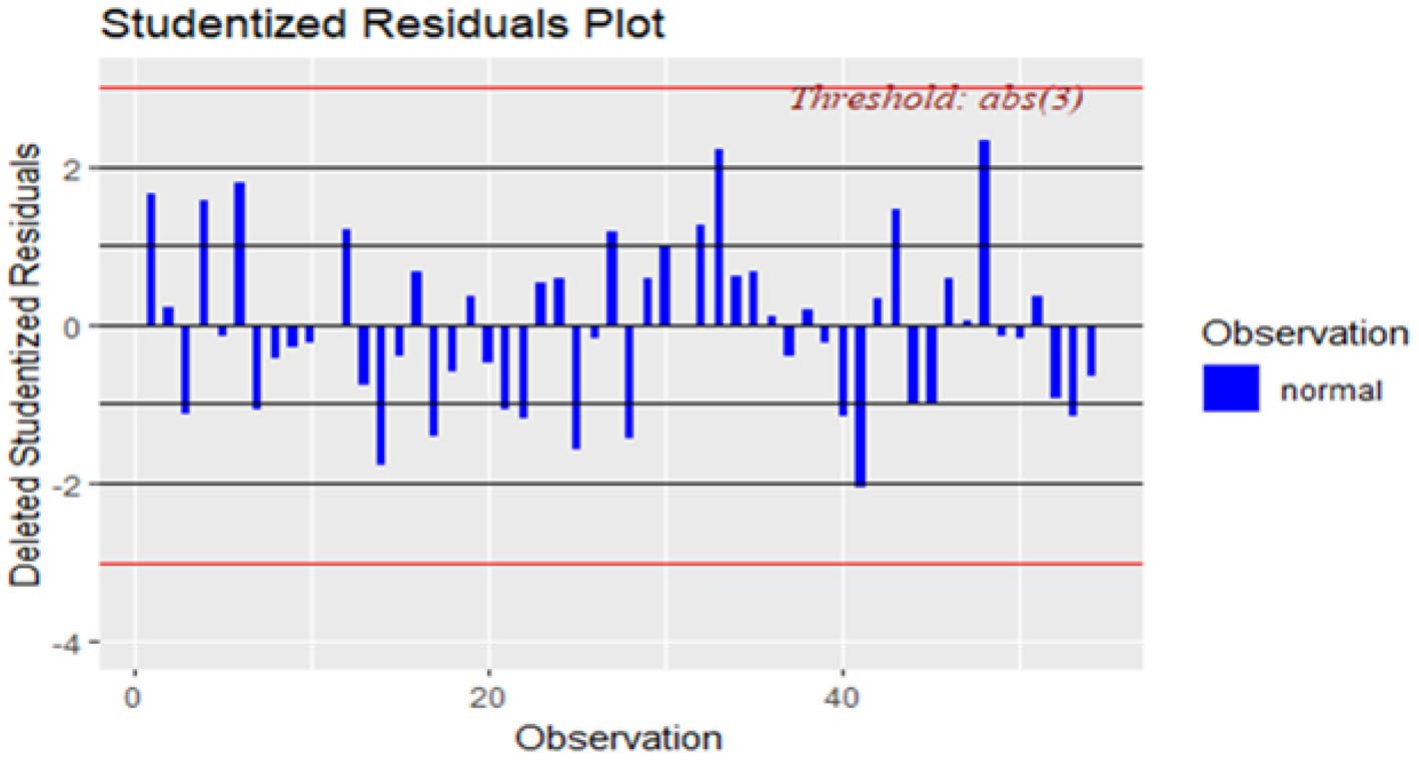
Studentized Residuals plot.

Kolmogorov–Smirnov test is used to test the normality assumption of the residuals, and a p-value of more than 10% is obtained, which clearly shows that there is no deviation from normality. The Normal QQ plot on Fig. 9 gives the same result as the Kolmogorov–Smirnov test. A diagnostic analysis of the residuals is conducted to investigate other assumptions of the regression model, including independence and the existence of outliers. Figure 10 shows residual vs fitted plot for checking the equality of the error variances. This plot does not show any pattern of heteroscedasticity, and the studentized residual plot on Fig. 11 does not show any outliers, all the residuals are in the normal range. A Durbin–Watson test is used to test the correlation among the residuals produced a test statistic of d = 1.68, and the 5% significance levels of the upper and the lower critical values are dL, 0.05 = 1.62 and dU, 0.05 = 1.71, respectively. Since 4–d is more than dU, 0.05, the test supports the claim that errors are not correlated.

### Locally weighted regression models (LWR)

**Figure 12:**
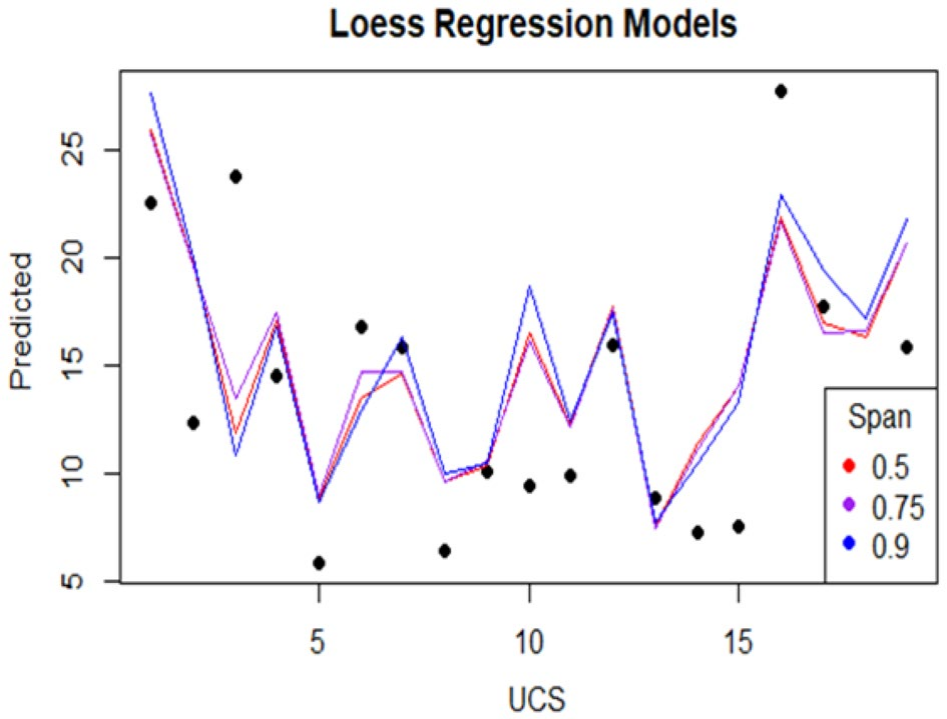
Plot of the fitted Loess models.

**Figure 13:**
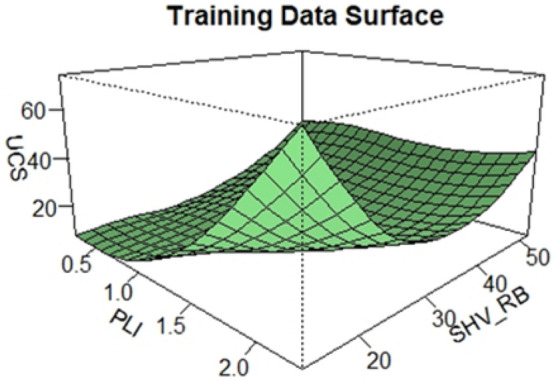
The surface plot of fitted Loess Model.

**Figure 14:**
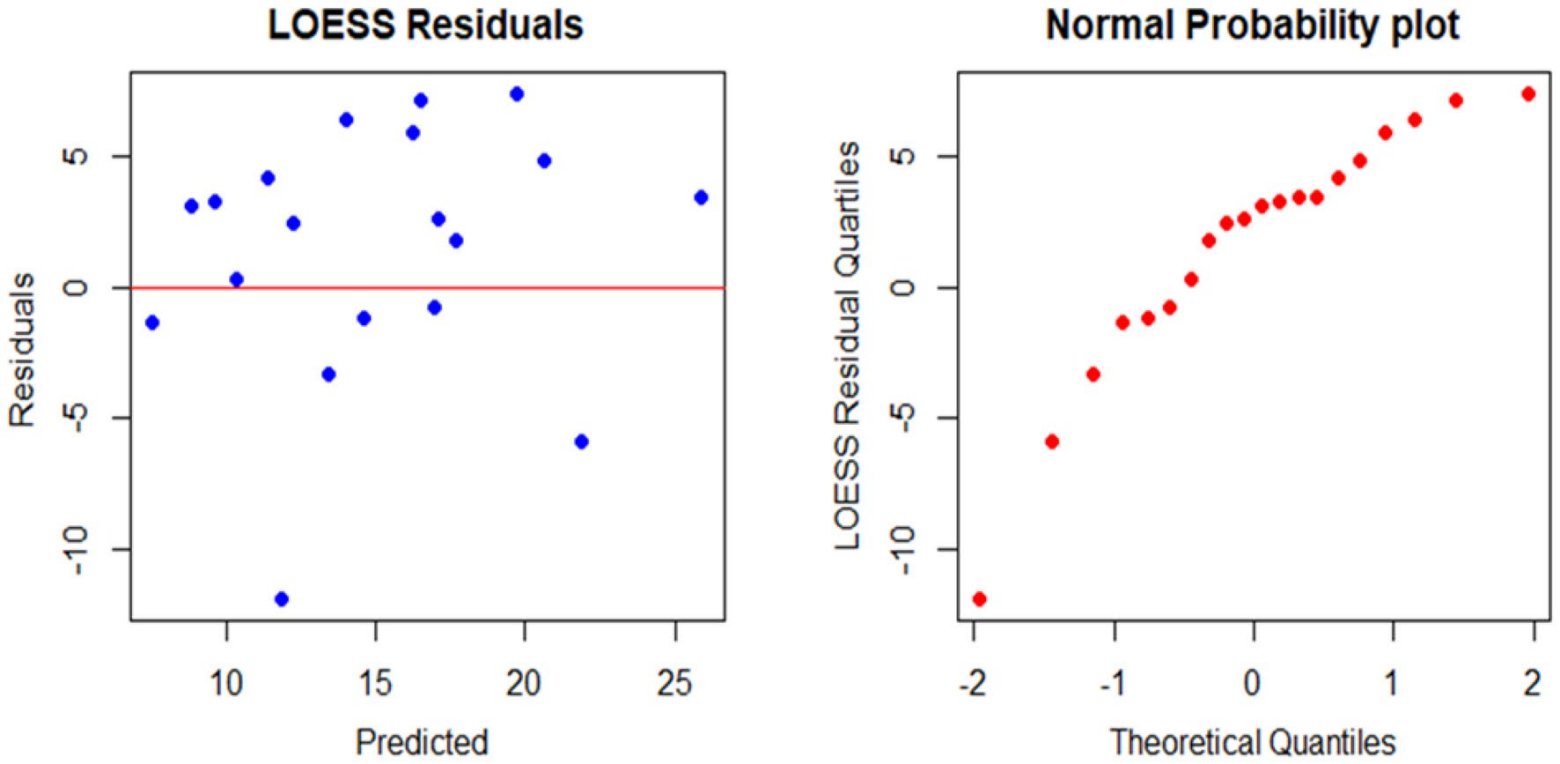
Residual plot of the Predicted Loess Model.

Firstly, visualizations of the training data is conducted to investigate the pattern of the data. The scatter plot on Fig. 2 and the surface plot on Fig. 3 of the training data both show that the relationship between the UCS and the PLI is clearly non-linear, but the UCS and the SHV_RB_ are linearly related. Secondly, a LOESS function with tri-cubic weight function is used to fit the data to a local regression model where the UCS is the dependent variable and the other two variables, PLI and SHV_RB_, are the predictors. Different spans and degrees of the LOESS function are used to search the best model for the estimation of the UCS. Based on the accuracy measures, RMSE and the MAE, the best LOESS model is degree 2 with a span of 0.90. Figure 12 displays the fitted LOESS model with spans of 0.5, 0.75, 90 whereas Fig. 13 shows the surface plot of the predicted model. The residual plots on Fig. 14 did not show any violations from the model assumptions.

To rank the competitiveness of the four models, R^2^, RMSE, MAE and A10-index accuracy measures are used to compare their performances., and the results of those measures are listed in Table 4.

**Table 4:** Model Results.

Model	R^2^	MAE	RMSE	A20
LWR	0.904	4.04	4.79	0.63
HYFIS	0.883	4.32	5.09	0.37
FMR	0.884	4.43	5.26	0.47
MLR	0.881	4.57	5.37	0.26

**Figure 15:**
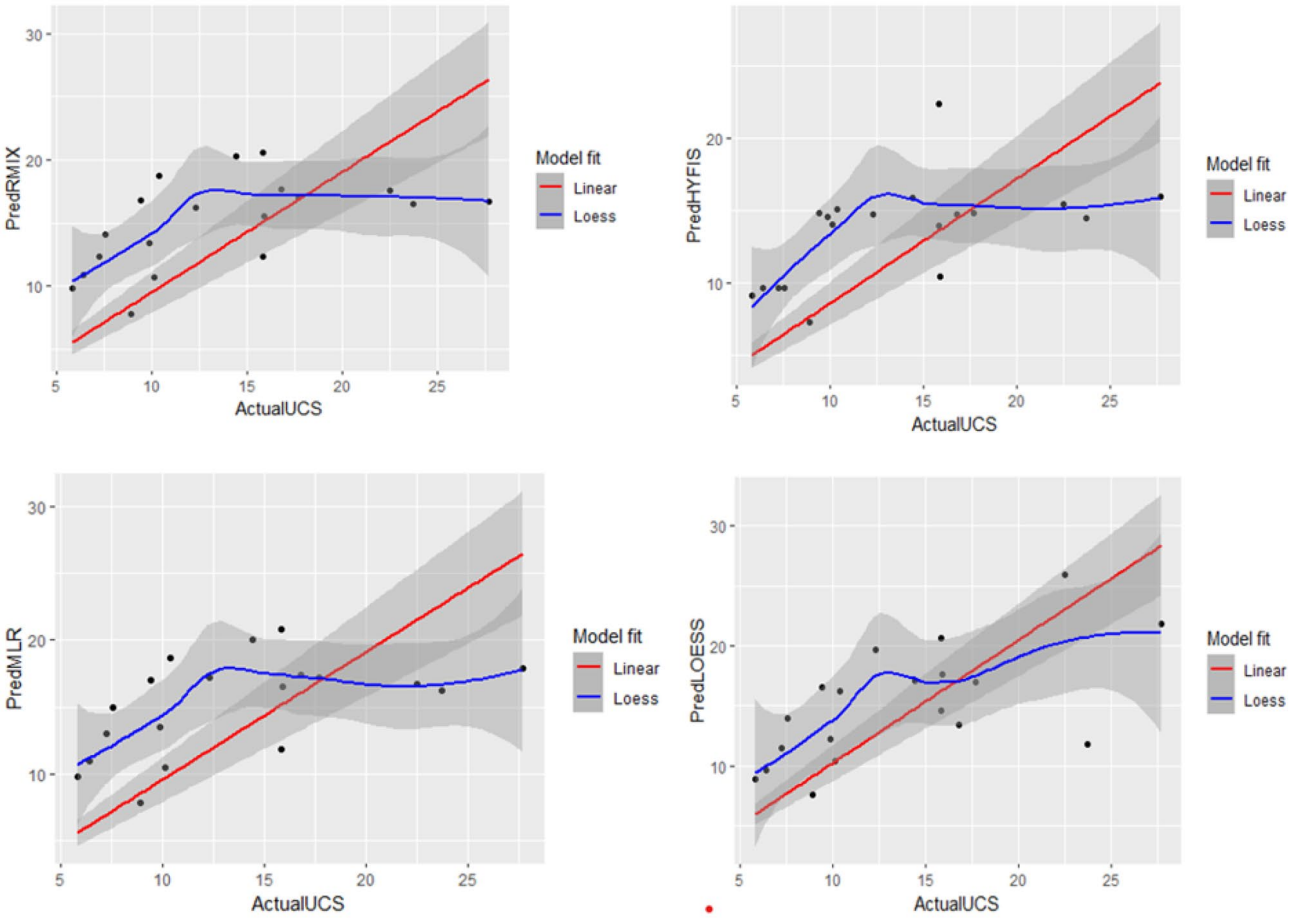
UCS predicted values.

All the above measures indicate that the LWR model outperformed all the other models. The HYFIS model has a slight advantage over the other two models, FMR and MLR. Figure 15 show the performances of the compared models.

**Figure 16:**
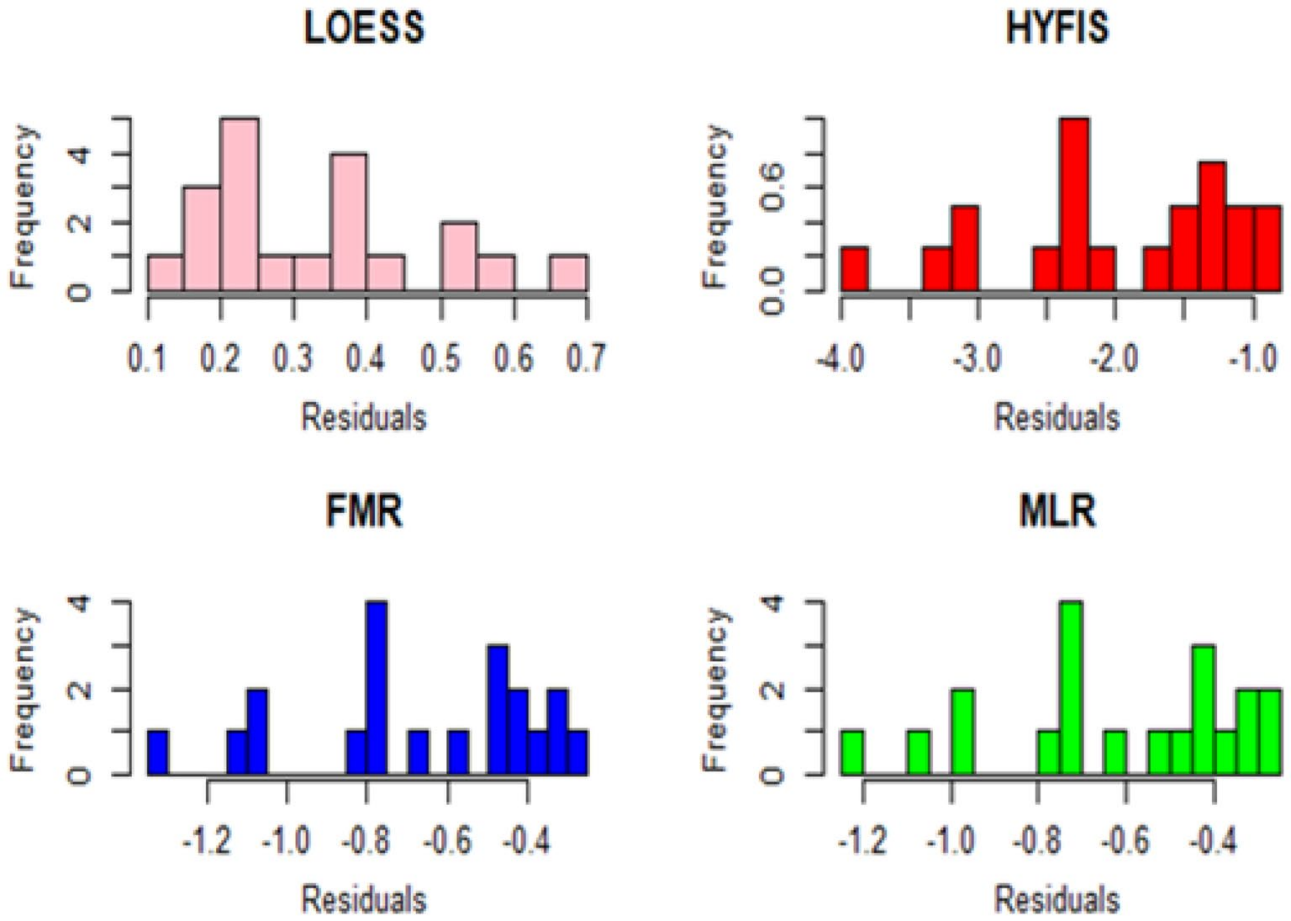
Residual histograms.

**Figure 17:**
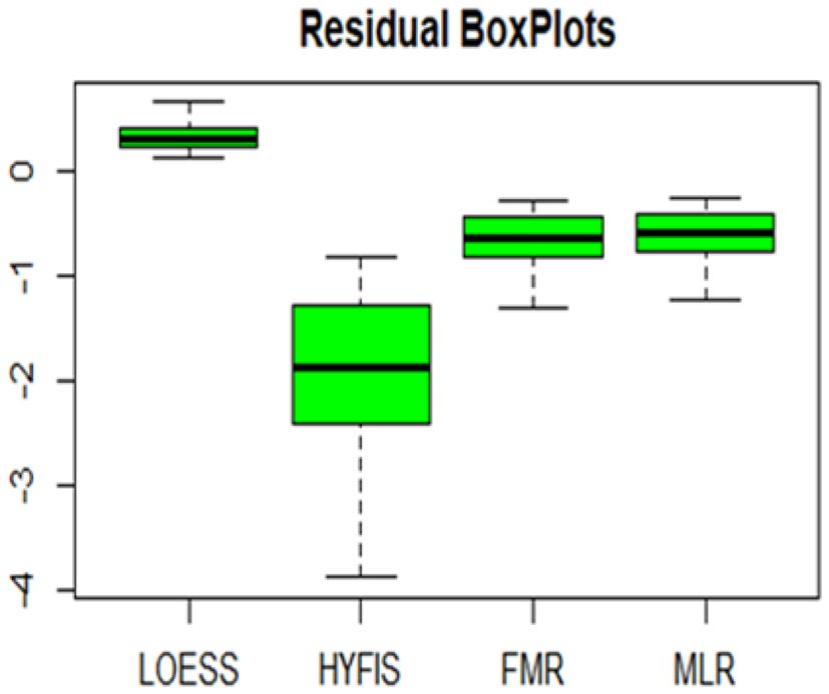
Residual boxplots.

Residual plots of the models are displayed in Figs. 16 and 17. Both the histograms and the boxplots do not deviate from symmetry.

## Limitations and future works

There are several limitations in this study. Firstly, the sample size was relatively small, and that has negatively affected the competitiveness of both the FMR and the HYFIS models, these models perform well for large sample sizes. Besides sample size, another limitation of the FMR is that it is not parsimonious, it usually has many independent parameters. This large number of the parameters inflate the information criteria like BIC and AIC which causes to take the edge off its competitiveness when comparing its performance to other parametric models if the population understudy is not heterogeneous. So, it deems necessary to use large samples in future studies to attain the advantages of these models, FMR and HYFIS, more competitive.

## Conclusion

In this study, different machine learning techniques, including hybrid fuzzy inference systems (HYFIS), finite mixture regression (FMR), locally weighted regression (LWR) and least squares multiple regression (MLR), are used for the prediction of uniaxial compressive strength (UCS) of evaporitic rocks from point load index (PLI) and Schmidt hammer tests (SHV_RB_). Different algorithms are implemented, including EM algorithm, Mamdani fuzzy rule structures, Gradient descent-based learning algorithm with multilayer perceptron (MLP), and the least squares. R^2^, RMSE, MAE and A20 accuracy measures are used to compare the performances of the competing models. The results of those measures for comparing the performances of those models are listed in Table 4.

## Data Availability

The datasets generated during and/or analyzed during the current study are available from the corresponding authors on reasonable request.
